# A Novel Particle Swarm Optimization Algorithm for Global Optimization

**DOI:** 10.1155/2016/9482073

**Published:** 2016-01-21

**Authors:** Chun-Feng Wang, Kui Liu

**Affiliations:** ^1^Department of Mathematics, Henan Normal University, Xinxiang 453007, China; ^2^Henan Engineering Laboratory for Big Data Statistical Analysis and Optimal Control, School of Mathematics and Information Sciences, Henan Normal University, Xinxiang 453007, China

## Abstract

Particle Swarm Optimization (PSO) is a recently developed optimization method, which has attracted interest of researchers in various areas due to its simplicity and effectiveness, and many variants have been proposed. In this paper, a novel Particle Swarm Optimization algorithm is presented, in which the information of the best neighbor of each particle and the best particle of the entire population in the current iteration is considered. Meanwhile, to avoid premature, an abandoned mechanism is used. Furthermore, for improving the global convergence speed of our algorithm, a chaotic search is adopted in the best solution of the current iteration. To verify the performance of our algorithm, standard test functions have been employed. The experimental results show that the algorithm is much more robust and efficient than some existing Particle Swarm Optimization algorithms.

## 1. Introduction

This paper considers the following global optimization problem:

(1) min fxs.t. x∈X,

where *x* is a continuous variable vector with domain *X* ⊂ *R* ^*n*^ defined by the bound constraint *l*_*j*_ ≤ *x*_*j*_ ≤ *u*_*j*_, *j* = 1,…, *n*. The function *f*(*x*) : *X* → *R* is a continuous real-valued function.

Many real-world problems, such as engineering and related areas, can be reduced to formulation (1). This problem usually has many local optima, so it is difficult to find its global optimum. For solving such problem, researchers have presented many methods during the past years, which can be divided into two groups: deterministic and stochastic algorithms. Most deterministic algorithms usually effective for unimodal functions have one global optimum and need gradient information. However, stochastic algorithms do not require any properties of the objective function. Therefore, more attention has been paid to stochastic algorithms recently, and many effective algorithms have been presented, including Simulated Annealing (SA) [1], Genetic Algorithm (GA) [2, 3], Differential Evolution (DE) [4], Particle Swarm Optimization (PSO) [5], Ant Colony Optimization (ACO) [6], Artificial Bee Colony (ABC) [7], and Harmony Search (HS) [8].

Among these stochastic algorithms, PSO is a population-based and intelligent method, which is inspired by the emergent motion of a flock of birds searching for food [5]. In PSO, a population of potential solutions is evolved through successive iterations. Since PSO algorithm has a number of desirable properties, including simplicity of implementation, scalability in dimension, and good empirical performance, it has been applied to solve many real-world problems, such as capacitor placement problem [9], short term load forecasting [10], soft sensor [11], the voltage stability of the electric power distribution systems [12, 13], the orbits of discrete chaotic dynamical systems towards desired target region [14], and the permutation flow-shop sequencing problem [15].

Although PSO algorithm has been applied successfully in solving many difficult optimization problems, it also has difficulties in keeping balance between exploration and exploitation when solving complex multimodal problems. In order to get a better performance for PSO algorithm, many variants of PSO have been developed. For example, by using random value of inertia weight, Eberhart and Shi proposed a modified PSO, which can track the optima in a dynamic environment [16]. By utilizing the success rate of the particles, a new adaptive inertia weight strategy was presented [17]. Through using Cauchy mutation, a hybrid PSO (HPSO) was developed [18]. In [19], a hybrid PSO with a wavelet mutation (HWPSO) was given, in which the mutation incorporates with a wavelet function. To avoid premature convergence, a novel parameter automation strategy is proposed [20]. Valdez et al. introduced an improved FPSO + FGA hybrid method by combining the advantages of PSO and GA [21]. Based on mutation operator and different local search techniques, a superior solution guided PSO (SSG-PSO) was developed [22]. By using the second personal best and the second global best particle, two modified PSO algorithms are presented [23]. In order to avoid being trapped in local optima in the convergence process, some other improved PSO have been proposed, such as crossover [24], orthogonal learning strategy [25], chaos [26], and elitist learning strategy [27].

In this paper, by utilizing the information of the the best neighbor of each particle and the best particle of the entire population in the current iteration, a new Particle Swarm Optimization algorithm is proposed, which is named NPSO. To avoid premature, an abandoned mechanism is presented in our algorithm. Furthermore, for improving the global convergence speed, a chaotic search is implemented in the best solution of each iteration.

The remainder of this paper is organized as follows.  Section 2 describes the original PSO. Then our proposed modifications to PSO are described in Section 3. Numerical results and discussions are presented in Section 4. Finally, some concluding remarks are provided in Section 5.

## 2. Particle Swarm Optimization (PSO)

Assume that the search space is *n*-dimensional; *M* denotes the size of the swarm population. In PSO, each particle *i*  (*i* = 1,…, *M*) has a position *x*_*i*_ = (*x*_*i*,1_, *x*_*i*,2_,…, *x*_*i*,*n*_) in the search space and a velocity *v*_*i*_ = (*v*_*i*,1_, *v*_*i*,2_,…, *v*_*i*,*n*_) to indicate its current state. A position *x*_*i*_ denotes a feasible solution. The position *x*_*i*_ and the velocity *v*_*i*_ are updated by the best position *p*_*i*_ = (*p*_*i*,1_, *p*_*i*,2_,…, *p*_*i*,*n*_) encountered by the particle so far and the best position *p*_*g*_ = (*p*_*g*,1_, *p*_*g*,2_,…, *p*_*g*,*n*_) found by the entire population of particles according to the following equations:

(2) vi,dt+1=ωvi,dt+c1r1pi,dt−xi,dt+c2r2pg,dt−xi,dt,xi,dt+1=xi,dt+vi,dt+1,

where *c*_1_ and *c*_2_ are two learning factors which control the influence of the social and cognitive components, *r*_*i*_  (*i* = 1,2) are random numbers in the range [0,1], and *ω* is the inertia weight, which ensures the convergence of the PSO algorithm and is decreased linearly.

## 3. The Novel PSO Algorithm (NPSO)

In the original PSO, since each particle moves in the search space guided only by its historical best solution *p*_*i*_ and the global best solution *p*_*g*_, it may get trapped in a local optimal solution when current global best solution in a local optimum and not easy for the particle escapes from it. To solve such problem, in this section, three improvement strategies are proposed.

### 3.1. The First Improvement Strategy

From (2), we observe that only the information of the historical best position *p*_*i*_ of each particle and the global best position *p*_*g*_ of all particles is utilized. As a matter of fact, the information of the best neighbor *p*_*N*_*i*__^best^ of the particle *i* may provide a better guidance than *p*_*i*_. The details are given as follows.

Firstly, we explain how to define the neighbors and to determine the best neighbor *p*_*N*_*i*__^best^ of the particle *i*. In order to define appropriate neighbors, different approaches could be used. In this paper, the neighbors of *x*_*i*_ are defined by using the mean Euclidean distance between *x*_*i*_ and the rest of solutions. Let *d*(*i*, *j*) be the Euclidean distance between *x*_*i*_ and *x*_*j*_ and let md_*i*_ be the mean Euclidean distance for *x*_*i*_. Then md_*i*_ can be computed as follows:

(3) $$\begin{array}{c} md_{i}=\frac{\sum_{j=1}^{M}d\left(i,j\right)}{M-1}. \end{array}$$

By (3), if *d*(*i*, *j*) < md_*i*_, then *x*_*j*_ could be accepted as a neighbor of *x*_*i*_.

In addition, we can also use a more general and flexible definition to determine a neighbor of *x*_*i*_:

(4) if  di,j≤r×mdi,  then  xj  is  a  neighbor  of  xi;  else  it  is  not.

If (4) is used, then a new parameter *r*  (*r* ≥ 0), which refers to the “neighbourhood radius,” will be added to the parameters of PSO. If *r* = 0, it turns to the standard PSO. With the value of *r* increasing, the neighborhood of *x*_*i*_ enlarges or its neighborhood shrinks as the value of *r* decreases. Once the neighbors are determined, we select the best position among the neighbors of *x*_*i*_ as the best neighbor *p*_*N*_*i*__^best^.

After determining the the best neighbor *p*_*N*_*i*__^best^, we give a new way moving for each particle. If *f*(*p*_*N*_*i*__^best^) < *f*(*p*_*i*_), then set

(5) vi,dt+1=ωvi,dt+c1r1pNi,dbestt−xi,dt+c2r2pg,dt−xi,dt.

If *f*(*p*_*N*_*i*__^best^) > *f*(*p*_*i*_), then set

(6) vi,dt+1=ωvi,dt+c1r1pi,dt−xi,dt+c2r2pg,dt−xi,dt.

After that, *x*_*i*_ moves to a new position by the following equation:

(7) $$\begin{array}{c} x_{i,d}\left(\;t+1\;\right)=x_{i,d}\left(\;t\;\right)+v_{i,d}\left(\;t+1\;\right). \end{array}$$

In our algorithm, it is obvious that before each particle moves, it first watches the region which is centered by itself, selects the best neighbour, and then uses (5)–(7) to generate the next position.

### 3.2. The Second Improvement Strategy

To avoid premature, in our algorithm, an abandoned mechanism is proposed.

Assume that “*L*” is a predetermined number, which will be used to determine whether the position *x*_*i*_ of the particle *i* should be abandoned. At the beginning, set *l*(*i*) = 0  (*i* = 1,…, *M*). With the implementation of the algorithm, if the position *x*_*i*_ was not improved, then set *l*(*i*) = *l*(*i*) + 1; else set *l*(*i*) = 0. If the position *x*_*i*_ can not be improved anymore when *L* is achieved, that is, *l*(*i*) = *L*, then the position will be abandoned and a new position *x*_new_ will replace it, which is generated by using the following equation:

(8) $$\begin{array}{c} x_{new}=\tau *p_{g}+\phi *\left(\;p_{g}-x_{i}\;\right), \end{array}$$

where *ϕ* is a random number in the range [−1,1] and *τ* is the inertia weight that controls the impact of the optimal solution at current iteration, which is increased linearly.

From (8), it can be seen that, in the early stage of the algorithm, *x*_new_ will be generated with a large randomness and in the late stage, it will be generated near the global best position *p*_*g*_.

### 3.3. The Third Improvement Strategy

To improve the global convergence of NPSO, a chaotic search operator is adopted. Next, we give the details.

Let *p*_*g*_ be the best solution of the current iteration. Firstly, utilize the following equation (9) to generate chaotic variable ch_*i*_:

(9) $$\begin{array}{c} ch_{i+1}=4*ch_{i}*\left(\;1-ch_{i}\;\right),\;1\leq i\leq K, \end{array}$$

where *K* is the length of chaotic sequence and ch_0_ ∈ (0,1) is a random number. Then map ch_*i*_ to a chaotic vector CH_*i*_ in the interval [*l*, *u*]:

(10) $$\begin{array}{c} CH_{i}=l+ch_{i}*\left(\;u-l\;\right),\;i=1,\ldots ,K, \end{array}$$

where *l* and *u* are the lower bound and upper bound of variable *x*, respectively. Finally, a new candidate solution ${\hat{x}}_{i}$ is obtained by the following equation:

(11) $$\begin{array}{c} {\hat{x}}_{i}=\left(\;1-\lambda \;\right)*p_{g}+\lambda *CH_{i},\;i=1,\ldots ,K, \end{array}$$

where *λ* is a shrinking factor, which is defined as follows:

(12) $$\begin{array}{c} \lambda =\frac{maxcycle-t+1}{maxcycle}, \end{array}$$

where maxcycle is the maximum number of iterations and *t* is the number of iterations.

By (11) and (12), it can be seen that *λ* will become smaller with the increase of evolutionary generation; that is, the local search range will become smaller with the process of evolution.

Based on the abovementioned explanation, the pseudocode of the NPSO algorithm is given in Algorithm 1.

## 4. Experimental Results and Discussion

### 4.1. Experiments 1

In this subsection, the performance of NPSO algorithm is compared to PSO algorithm by evaluating convergence and best solution found for 14 benchmark functions, where *f*_7_–*f*_11_ are shifted functions and *f*_12_–*f*_14_ are rotated functions. The characteristics, dimensions, initial range, and formulations of these functions are listed in Table 1. *z* is **x** − **o** for *f*_7_–*f*_11_ and (**x** − **o**)*∗* **M** for *f*_12_–*f*_14_; **o** = (*o*_1_, *o*_2_,…, *o*_*n*_) is the shifted global optimum; **M** is the *n* × *n* orthogonal matrix.

The proposed algorithm NPSO and PSO are coded in Matlab 7.0, and the experiments' platform is a personal computer with Pentium 4, 3.06 GHz CPU, 512 M memory, and Windows XP.

**Figure 1:**
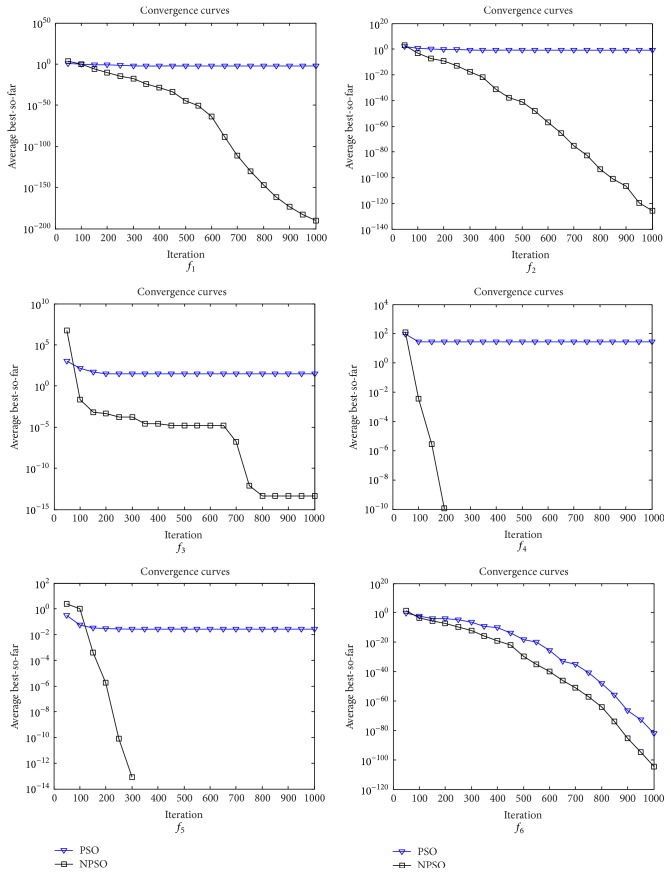
Convergence rates on test functions.

The parameters of algorithms are given as follows. The common parameters are the dimension *n* = 30, the population size *M* = 30, the maximum number of iterations maxcycle = 2000, *ω*_min_ = 0.4, *ω*_max_ = 0.9, *v*_min_ = 0.4, and *v*_max_ = 0.9. NPSO settings are as follows: the length *K* of chaotic sequence and the limit *L* of a position abandoned are set to 5; *r* = 0.01. Each experiment is repeated 30 times independently. The global minimums (Min), maximum number of iterations (Max iteration), mean best values (Mean), and standard deviations (SD) are given in Table 2. To show the convergence speed of PSO and NPSO more clearly, the convergence graphs of PSO and NPSO are shown in Figure 1.

From Table 2, it can be seen that the NPSO performs better than PSO on all test functions. From Figure 1, it can be seen that the convergence speed of NPSO is more fast than PSO.

### 4.2. Experiments 2

In this subsection, to further test the efficiency of NPSO, it is compared with other five algorithms, that is, CPSO [28], CLPSO [29], FIPS [30], Frankenstein [31], and AIWPSO [17].

Twelve benchmark functions are used for the comparison. The characteristics, dimensions, initial range, and formulations of these functions are listed in Table 3.

In order to make a fair comparison, the maximum number of function evaluations (maxFEs) is set to 2*e*5 for all algorithms; the population size is 20. The other parameters for NPSO are set as in Experiments 1. The comparison results are presented in Table 4. For the sake of convenience and reliability, except for the NPSO algorithm, the rest of results reported here are taken directly from the literature [17].

From Table 4, it can be seen that NPSO is significantly better than the other five algorithms on almost all the test functions, except for the function *f*_11_.

## 5. Conclusion

In this paper, by utilizing the information of the best neighbor of each particle and the best solution in the current iteration, we presented a new move equation. After that, based on the other two improvement strategies, a novel Particle Swarm Optimization algorithm NPSO was proposed. The performance of NPSO was compared with the standard PSO and other five variants of PSO. The results showed that NPSO presents promising results for considered problems.

In the future, the adaption of the parameters in NPSO can be studied to improve its performance.

## Figures and Tables

**Algorithm 1:**
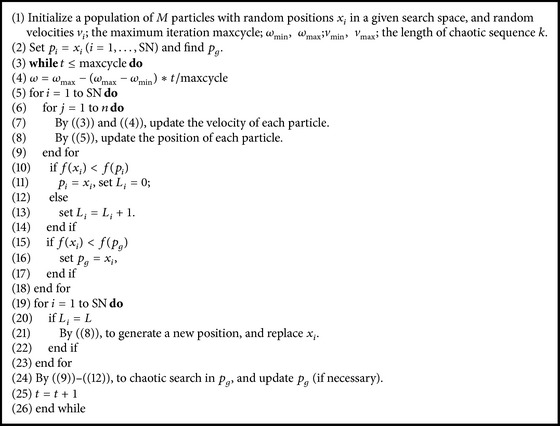
Framework of NPSO. Remark: in our algorithm, for simplicity, in (8), we can set *τ* = 1 − *ω*, which can meet our needs.

**Table 1:** Benchmark functions used in experiments.

Functions	Dimension	C	Range	Optimal value
$f_{1}=\overset{n}{\underset{i=1}{\sum }}x_{i}^{2}$	30	US	[−100,100]	0
$f_{2}=\overset{n}{\underset{i=1}{\sum }}ix_{i}^{2}$	30	US	[−10,10]	0
$f_{3}=\overset{n-1}{\underset{i=1}{\sum }}\left[100{\left(x_{i+1}-x_{i}^{2}\right)}^{2}+{\left(x_{i}-1\right)}^{2}\right]$	30	UN	[−30,30]	0
$f_{4}=\overset{n}{\underset{i=1}{\sum }}\left(x_{i}^{2}-10\cos\left(2\pi x_{i}\right)+10\right)$	30	MS	[−5.12,5.12]	0
$f_{5}=\frac{1}{4000}\overset{n}{\underset{i=1}{\sum }}x_{i}^{2}-\prod_{i=1}^{n}\cos\left(\frac{x_{i}}{\sqrt{i}}\right)+1$	30	MS	[−600,600]	0
$f_{6}=\overset{n}{\underset{i=1}{\sum }}\left|x_{i}\right|+\prod_{i=1}^{n}\left|x_{i}\right|$	30	UN	[−10,10]	0
$f_{7}=-20\exp\left(-0.2*\sqrt{\overset{n}{\underset{i=1}{\sum }}\frac{z_{i}^{2}}{n}}\right)$	30	MN	[−32,32]	0
$-\exp\left(\overset{n}{\underset{i=1}{\sum }}‍\cos\left(\frac{2\pi z_{i}}{n}\right)\right)+20+e$				
$f_{8}=\frac{1}{4000}\overset{n}{\underset{i=1}{\sum }}z_{i}^{2}-\prod_{i=1}^{n}\cos\left(\frac{z_{i}}{\sqrt{i}}\right)+1$	30	MS	[−600,600]	0
$f_{9}=\overset{n}{\underset{i=1}{\sum }}z_{i}^{2}$	30	US	[−100,100]	0
$f_{10}=\overset{n}{\underset{i=1}{\sum }}{\left(\overset{i}{\underset{j=1}{\sum }}z_{j}\right)}^{2}$	30	UN	[−100,100]	0
$f_{11}=\overset{n}{\underset{i=1}{\sum }}\left(z_{i}^{2}-10\cos\left(2\pi z_{i}\right)+10\right)$	30	MS	[−5.12,5.12]	0
$f_{12}=\overset{n}{\underset{i=1}{\sum }}{\left(\overset{i}{\underset{j=1}{\sum }}z_{j}\right)}^{2}-450$	30	UN	[−100,100]	−450
$f_{13}=\overset{n}{\underset{i=1}{\sum }}(z_{i}^{2}-10\;cos\;(2\pi z_{i})+10)-330$	30	MS	[−5.12,5.12]	−330
$f_{14}=\overset{n}{\underset{i=1}{\sum }}\left(\overset{k_{max}}{\underset{k=0}{\sum }}\left[a^{k}\cos\left(2*\pi b^{k}\left(z_{i}+0.5\right)\right)\right]\right)$	30	US	[−0.5,0.5]	90
$-n\overset{k_{max}}{\underset{k=0}{\sum }}\left[a^{k}cos\;\left(2\pi b^{k}0.5\right)\right]+90,\;a=0.5,\;b=3,\;k_{max}=20$				

C: characteristic, U: unimodal, M: multimodal, N: nonseparable, and S: separable.

**Table 2:** NPSO performance comparison with PSO.

Function	Max iteration	Algorithm	Mean	SD	Min
*f*_1_	1000	PSO	5.06*e* − 003	1.26*e* − 003	4.10*e* − 003
		NPSO	5.66*e* − 141	9.80*e* − 141	1.97*e* − 198
*f*_2_	1000	PSO	1.81*e* − 001	1.65*e* − 001	3.08*e* − 002
		NPSO	2.41*e* − 129	4.17*e* − 129	2.91*e* − 201
*f*_3_	1000	PSO	2.95*e* + 001	3.55*e* − 001	2.91*e* + 001
		NPSO	2.217*e* − 005	3.48*e* − 005	6.81*e* − 013
*f*_4_	1000	PSO	2.90*e* + 001	0	2.90*e* + 001
		NPSO	0	0	0
*f*_5_	1000	PSO	4.97*e* − 004	4.65*e* − 004	1.91*e* − 004
		NPSO	0	0	0
*f*_6_	1000	PSO	4.55*e* − 096	3.06*e* − 096	1.12*e* − 096
		NPSO	4.49*e* − 104	7.78*e* − 104	7.40*e* − 117
*f*_7_	1000	PSO	2.03*e* + 001	1.22*e* − 002	2.02*e* + 001
		NPSO	3.29*e* − 007	3.76*e* − 007	1.75*e* − 008
*f*_8_	1000	PSO	1.054*e* + 002	9.24*e* − 002	1.053*e* + 002
		NPSO	3.68*e* − 009	6.27*e* − 009	6.00*e* − 012
*f*_9_	1000	PSO	2.32*e* + 004	3.03*e* + 002	2.30*e* + 004
		NPSO	6.30*e* − 012	1.04*e* − 011	1.29*e* − 014
*f*_10_	1000	PSO	3.84*e* + 007	7.18*e* + 004	3.83*e* + 007
		NPSO	3.79*e* − 009	6.41*e* − 009	3.29*e* − 012
*f*_11_	1000	PSO	2.44*e* + 001	1.25*e* + 000	2.33*e* + 001
		NPSO	1.66*e* − 004	2.03*e* − 004	1.25*e* − 009
*f*_12_	1000	PSO	8.34*e* + 002	1.05*e* + 003	1.22*e* + 002
		NPSO	−4.499*e* + 002	4.25*e* − 004	−450
*f*_13_	1000	PSO	−2.59*e* + 002	1.25*e* + 001	−2.69*e* + 002
		NPSO	−330	0	−330
*f*_14_	1000	PSO	1.24*e* + 002	1.02*e* + 000	1.23*e* + 002
		NPSO	9.001*e* + 001	2.23*e* − 002	90

**Table 3:** Benchmark functions used in experiments.

Functions	Dimension (*n*)	C	Range	Optimal value
$f_{1}=\overset{n}{\underset{i=1}{\sum }}x_{i}^{2}$	30	US	[−100,100]	0
$f_{2}=\overset{n}{\underset{i=1}{\sum }}\left|x_{i}\right|+\prod_{i=1}^{n}\left|x_{i}\right|$	30	UN	[−10,10]	0
$f_{3}=\overset{n}{\underset{i=1}{\sum }}{\left(\overset{i}{\underset{j=1}{\sum }}x_{j}\right)}^{2}$	30	UN	[−100,100]	0
$f_{4}=\overset{n}{\underset{i=1}{\sum }}ix_{i}^{4}+random\left[0,1\right)$	30	MS	[−1.28,1.28]	0
$f_{5}=\overset{n-1}{\underset{i=1}{\sum }}\left[100{\left(x_{i+1}-x_{i}^{2}\right)}^{2}+{\left(x_{i}-1\right)}^{2}\right]$	30	UN	[−30,30]	0
$f_{6}=\overset{n}{\underset{i=1}{\sum }}{\left(\left\lfloor x_{i}+0.5\right\rfloor \right)}^{2}$	30	US	[−1.28,1.28]	0
$f_{7}=\overset{n}{\underset{i=1}{\sum }}\left(x_{i}^{2}-10\cos\left(2\pi x_{i}\right)+10\right)$	30	MS	[−5.12,5.12]	0
$f_{8}=\overset{n}{\underset{i=1}{\sum }}\left(y_{i}^{2}-10\cos\;\left(2\pi y_{i}\right)+10\right)$,	30	US	[−5.12,5.12]	0
if $|x_{i}|<\frac{1}{2},\;y_{i}=x_{i};\;$else $y_{i}=\frac{round\;(2x_{i})}{2}$				
$f_{9}=-20\;exp\left(-0.2*\sqrt{\overset{n}{\underset{i=1}{\sum }}\frac{x_{i}^{2}}{n}}\right)$	30	MN	[−32,32]	0
$-exp\left(\overset{n}{\underset{i=1}{\sum }}cos\left(\frac{2\pi x_{i}}{n}\right)\right)+20+e$				
$f_{10}=\frac{1}{4000}\overset{n}{\underset{i=1}{\sum }}x_{i}^{2}-\prod_{i=1}^{n}\cos\;\left(\frac{x_{i}}{\sqrt{i}}\right)+1$	30	MS	[−600,600]	0
$f_{11}=\overset{n}{\underset{i=1}{\sum }}-x_{i}*\sin\left(\sqrt{\left|x\right|}\right)$	30	MS	[−500,500]	418.98288*∗n*
$f_{12}=\overset{n}{\underset{i=1}{\sum }}x_{i}^{2}$	30	US	[−10,190]	0

C: characteristic, U: unimodal, M: multimodal, N: nonseparable, and S: separable.

**Table 4:** The mean and standard deviation of the best solutions of six PSO variants on 12 test problems in 200,000 function evaluations.

	*f*_1_	*f*_2_	*f*_3_
CPSO	5.146*e* − 013 (7.7588*e* − 025)	1.2534*e* − 007 (1.1791*e* − 014)	1.8889*e* + 003 (9.9106*e* + 006)
CLPSO	4.894*e* − 039 (6.7814*e* − 078)	8.8677*e* − 024 (7.9008*e* − 049)	1.9217*e* + 002 (3.8433*e* + 003)
FIPS	4.588*e* − 027 (1.9577*e* − 053)	2.3239*e* − 016 (1.1406*e* − 032)	9.4634*e* + 000 (2.5976*e* + 001)
Frankenstein	2.409*e* − 016 (2.0047*e* − 031)	1.5804*e* − 011 (1.0301*e* − 022)	1.7315*e* + 002 (9.1577*e* + 003)
AIWPSO	3.370*e* − 134 (5.1722*e* − 267)	1.6534*e* − 062 (7.7348*e* − 123)	1.9570*e* − 010 (1.2012*e* − 019)
NPSO	6.597**e** − 217 (1.632**e** − 235)	2.5016**e** − 109 (4.3331**e** − 109)	5.4552**e** − 193 (9.2401**e** − 198)

	*f*_4_	*f*_5_	*f*_6_

CPSO	1.0764*e* − 002 (2.7698*e* − 005)	8.2648*e* − 001 (2.3449*e* + 00)	0.0 (0.0)
CLPSO	4.0642*e* − 003 (9.6184*e* − 007)	1.3217*e* + 001 (2.1480*e* + 002)	0.0 (0.0)
FIPS	3.3047*e* − 003 (8.6680*e* − 007)	2.6714*e* + 001 (2.0025*e* + 002)	0.0 (0.0)
Frankenstein	4.1690*e* − 003 (2.4012*e* − 006)	2.8156*e* + 001 (2.3132*e* + 002)	0.0 (0.0)
AIWPSO	5.5241*e* − 003 (1.5358*e* − 005)	2.5003*e* + 000 (1.5978*e* + 001)	0.0 (0.0)
NPSO	8.4591**e** − 006 (8.4932**e** − 006)	3.0621**e** − 006 (4.0283**e** − 006)	0.0 (0.0)

	*f*_7_	*f*_8_	*f*_9_

CPSO	3.6007*e* − 013 (1.5035*e* − 024)	5.3717*e* − 013 (5.9437*e* − 024)	1.6091*e* − 007 (7.8608*e* − 014)
CLPSO	0.0 (0.0)	1.3333*e* − 001 (1.1954*e* − 001)	9.2371*e* − 015 (6.6156*e* − 030)
FIPS	5.8502*e* + 001 (1.9185*e* + 002)	6.1883*e* + 001 (1.4013*e* + 002)	1.3856*e* − 014 (2.3227*e* − 029)
Frankenstein	7.3836*e* + 001 (3.7055*e* + 002)	7.0347*e* + 001 (2.9600*e* + 002)	2.1792*e* − 009 (1.7187*e* − 018)
AIWPSO	1.6583*e* − 001 (2.1051*e* − 001)	1.1842*e* − 016 (4.2073*e* − 031)	6.9870*e* − 015 (4.2073*e* − 031)
NPSO	0.0 (0.0)	4.0407**e** − 215 (1.5575**e** − 220)	−8.8817**e** − 016 (0.0)

	*f*_10_	*f*_11_	*f*_12_

CPSO	2.1245*e* − 002 (6.3144*e* − 004)	−1.2127*e* + 004 (3.3795*e* + 004)	5.4282*e* − 014 (8.2868*e* − 027)
CLPSO	0.0 (0.0)	−1.2546*e* + 004 (4.2567*e* + 003)	9.9748*e* − 039 (3.7661*e* − 084)
FIPS	2.4776*e* − 004 (1.8266*e* − 006)	−1.1052*e* + 004 (9.4421*e* + 005)	2.6033*e* + 002 (2.1785*e* + 004)
Frankenstein	1.4736*e* − 003 (1.2846*e* − 005)	−1.1221*e* + 004 (2.7708*e* + 005)	5.1953*e* + 004 (1.1136*e* + 009)
AIWPSO	2.8524*e* − 002 (7.6640*e* − 004)	−1.2569**e** + 004 (1.1409**e** − 025)	1.8317*e* − 137 (3.4534*e* − 273)
NPSO	0.0 (0.0)	− 1.2569*e* + 004 (2.5299*e* − 007)	2.2740**e** − 198 (2.6952**e** − 186)
